# Bowen’s disease of the penile shaft presenting as a pigmented macule: dermoscopy, reflectance confocal microscopy and histopathological correlation^☆^^☆☆^

**DOI:** 10.1016/j.abd.2020.10.009

**Published:** 2021-07-18

**Authors:** Francesco Lacarrubba, Anna Elisa Verzì, Rosario Caltabiano, Giuseppe Micali

**Affiliations:** Dermatology Clinic, University of Catania, Catania, Italy

**Keywords:** Bowen's disease, Carcinoma, squamous cell, Dermoscopy, Microscopy, confocal, Papillomavirus infections

## Abstract

The penile localization of pigmented Bowen’s disease has been rarely reported and has been mostly related to human papillomavirus infection. Early diagnosis and treatment are important to prevent progression to invasive squamous cell carcinoma. However, diagnosis can be challenging because it may be difficult to distinguish from melanoma, even using dermoscopy. Reflectance confocal microscopy may be useful in suggesting the bedside diagnosis before the histopathological confirmation. A case of penile pigmented Bowen’s disease is described along with its dermoscopy and reflectance confocal microscopy findings and their correlation with histopathology.

## Case report

**Figure 1:**
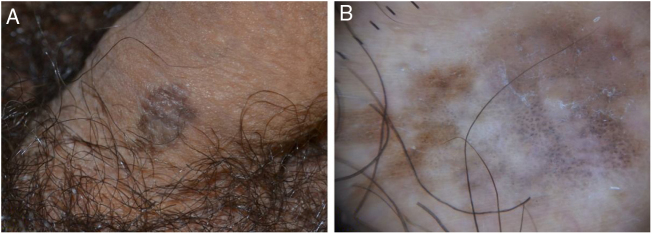
(A), roundish, irregularly pigmented macule of the penile shaft. (B), polarized light dermoscopy showing an irregular brownish pigmentation, multiple brown-grey dots and globules, and structureless whitish areas.

**Figure 2:**
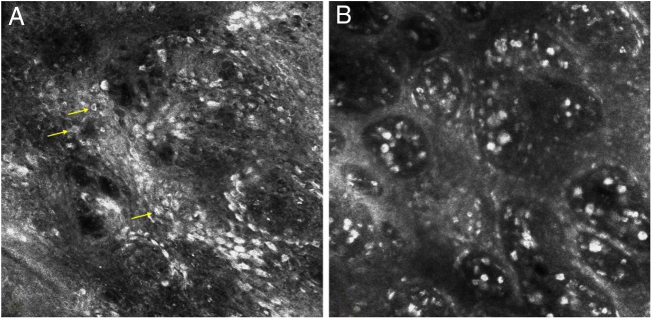
Reflectance confocal microscopy showing (A), irregular and disarranged honeycomb pattern and several bright, roundish nucleated cells with a targetoid appearance (arrows) in the epidermis. (B), edged papillae and multiple bright plump cells in the papillary dermis.

A 34-year-old Caucasian man presented with a brownish lesion of the penile shaft that appeared about 2 years earlier and slowly enlarging. Clinical examination revealed the presence of a roundish, irregularly pigmented macule measuring 1.8 × 1.2 cm (Fig. 1A). Polarized light dermoscopy (Illuco IDS-1100®, Tre T Medical, Camposano, Italy) showed an irregular brownish pigmentation, multiple brown-grey dots, and globules, and structureless whitish areas (Fig. 1B). Handheld Reflectance Confocal Microscopy (RCM) (Vivascope 3000®, Mavig GmbH, Munich, Germany) showed parakeratosis, an irregular and disarranged honeycomb pattern, and sparse bright dendritic cells in the spinous-granular layer; moreover, several bright, roundish nucleated cells with a targetoid appearance were visible throughout the epidermis (Fig. 2A). The Dermo-Epidermal Junction (DEJ) was preserved with the presence of well-defined dermal papillae rimmed by hyperrefractile rings (“edged papillae”). In the papillary dermis, an increased blood flow and multiple bright plump cells were detected (Fig. 2B).

**Figure 3:**
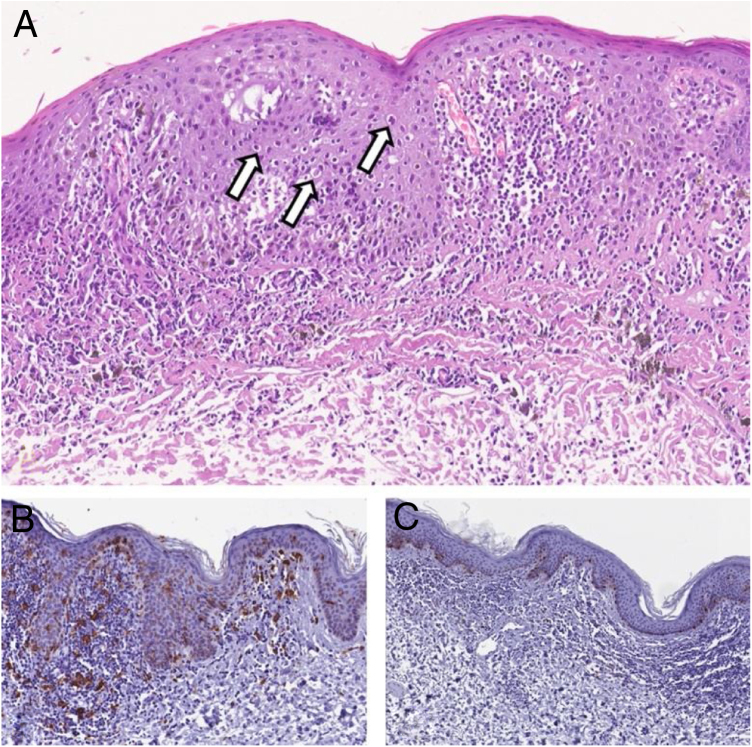
(A), Histopathology showing in the epidermis atypical features consisting of abnormal maturation of keratinocytes, prominent nucleoli, and many dyskeratotic cells (arrows). In the superficial dermis, ectatic vessels, a moderate lymphohistiocytic infiltrate and melanophages may be observed (Hematoxyline & eosin, ×150). (B), At immunohistochemistry CD1a + Langerhans cells, may be observed (Immunohistochemical, ×200). (C), Immunohistochemical staining for Melan A showed no atypical melanocytes. Hyperpigmentation of keratinocytes of the basal layer is evident (Immunohistochemical, ×150).

The lesion was excised, and histopathological examination revealed hyperkeratosis, parakeratosis, acanthosis, abnormal maturation of keratinocytes, prominent nucleoli, and dyskeratotic cells in all layers of the epithelium. In the superficial dermis, ectatic vessels, a moderate lymphohistiocytic infiltrate and melanophages were observed (Fig. 3A). Immunohistochemistry showed that the bright dendritic cells observed at RCM were CD1a positive Langerhans cells, while staining for Melan A showed no atypical melanocytes and hyperpigmentation of keratinocytes of the basal layer (Fig. 3B and 3C); p16 immunostaining, indicative of high-risk Human Papillomavirus (HPV) infection, was positive.

The final diagnosis was HPV-associated with pigmented Bowen’s disease.

## Discussion

Bowen’s disease represents an “in situ” variant of cutaneous squamous cell carcinoma. Pigmented Bowen’s disease is a rare subtype accounting for 2%–5% of all cases.^1^, ^2^ It manifests as a slow-growing, well-defined, hyperpigmented macule, with a flat, velvety, or scaling surface and is a known melanoma simulator.^1^, ^2^ The penile localization of pigmented Bowen’s disease has been rarely reported and has been mostly related to HPV infection.

The dermoscopic features of penile pigmented Bowen’s disease have been reported to be similar to those observed outside the genital area: irregular brownish pigmentation, structureless areas (blue/black, hypopigmented, pink-grey, or skin-colored), and brown-grey dots/globules.^3^, ^4^, ^5^, ^6^ Glomerular vessels may also be observed. Dermoscopy may help in the differential diagnosis with other lesions that may simulate pigmented Bowen’s disease such as melanosis, flat seborrheic keratosis, and superficial basal cell carcinoma, but it cannot always rule out melanoma as it may share similar features.

Some studies reported the RCM features of non-genital pigmented Bowen’s disease and their histopathological correlations.^2^, ^7^, ^8^, ^9^ They mainly consist of parakeratosis, irregular honeycomb pattern (indicative of keratinocyte atypia and pleomorphism), edged papillae (due to hyperpigmentation of keratinocytes of the basal layer), and coiled vessels in the dermal papillae. Other observed findings are represented by round nucleated cells often with a targetoid appearance throughout the epidermis (corresponding to dyskeratotic keratinocytes), intraepidermal hyperreflective dendritic, spindle-shaped cells (corresponding to Langerhans cells at immunostaining), and plump bright cells in the papillary dermis (corresponding to melanophages).^2^, ^7^, ^8^, ^9^ It must be highlighted that the dendritic cells may represent a confounder finding as they may also correspond to atypical melanocytes and could lead to the wrong diagnosis of melanoma,^9^ and that the differential diagnosis of melanophages with pigmented cells of Bowen’s disease may not be easy due to the epidermis disruption and tumor cell heavy pigmentation.

In a previously reported case of non-pigmented in situ squamous cell carcinoma of the penis, RCM showed a disarranged pattern of the epithelium with many hyper-refractive dendritic cells.^10^ To the best our knowledge, the present case represents the first RCM description of penile pigmented Bowen’s disease. The same features of those previously described in non-genital pigmented Bowen’s disease confirmed the value of RCM in addressing the correct diagnosis by narrow down the differential diagnosis with clinically similar benign and malignant pigmented lesions, including melanoma.^2^

In conclusion, early diagnosis and treatment of penile Bowen’s disease are important to prevent progression to invasive squamous cell carcinoma, which may occur in up to 30% of cases, and the need for extensive mutilating surgery.^5^ However, Bowen’s disease diagnosis can be challenging because it may be difficult to distinguish from melanoma, even using dermoscopy. RCM may be useful in suggesting the bedside diagnosis before the histological confirmation, although further studies on larger case series are needed to validate the sensitivity and specificity of this method.

## Financial support

This work was supported by the University of Catania, Department of General Surgery and Medical-Surgical Specialties - Research Program 2016-2018, P.I. Prof. Giuseppe Micali.

## Authors' contributions

Francesco Lacarrubba: Intellectual contribution in conceiving and planning the study; collecting, analyzing, and interpreting data; writing or critically reviewing the manuscript; approving its final version.

Anna Elisa Verzì: Intellectual contribution in conceiving and planning the study; collecting, analyzing, and interpreting data; writing or critically reviewing the manuscript; approving its final version.

Rosario Caltabiano: Intellectual contribution in conceiving and planning the study; collecting, analyzing, and interpreting data; writing or critically reviewing the manuscript; approving its final version.

Giuseppe Micali: Intellectual contribution in conceiving and planning the study; collecting, analyzing, and interpreting data; writing or critically reviewing the manuscript; approving its final version.

## Conflicts of interest

None declared.
